# Febrile neutropenia in pediatric acquired aplastic anemia: a 20-year analysis of infections and mortality

**DOI:** 10.1007/s00277-026-06874-9

**Published:** 2026-05-25

**Authors:** Gulhadiye Avcu, Nihal Karadas, Aysha Gadashova, Cihan Onder, Asli Arslan, Zumrut Sahbudak Bal, Sohret Aydemir, Dilek Yesim Metin, Deniz Yilmaz Karapinar

**Affiliations:** 1 Faculty of Medicine, Department of Pediatrics, Division of Pediatric Infectious Diseases, Ege University, Izmir, Turkey; 2 Faculty of Medicine, Department of Pediatrics, Division of Pediatric Hematology, Ege University, Izmir, Turkey; 3 Faculty of Medicine, Department of Medical Microbiology, Ege University, Izmir, Turkey; 4 Faculty of Medicine, Department of Medical Microbiology, Division of Mycology, Ege University, Izmir, Turkey

**Keywords:** Acquired aplastic anemia, Febrile neutropenia, Infection, Survival, Children

## Abstract

Acquired aplastic anemia (AA) is a life-threatening bone marrow failure disorder, and febrile neutropenia (FN) episodes in children with AA pose significant risks due to infections. The study aimed to investigate the incidence, types, and outcomes of infections during FN episodes in pediatric AA patients. This single-centre retrospective cohort study included 39 pediatric patients with AA diagnosed between January 2004 and January 2024. Data from medical records, including clinical characteristics, laboratory findings, immunosuppressive therapy details, microbiological results, and infection outcomes, were analyzed. Infection sources, pathogens, and outcomes were assessed, and statistical analysis was performed to identify risk factors for infections and mortality. Twenty-five patients were male (64.1%), with a median age of 12,05 years (interquartile range : 6 to 14,94y). A total of 152 FN episodes were evaluated, with 87.1% of patients experiencing at least one episode [median, 4.15 (range:1–15)]. 58 (38.1%) episodes were classified as microbiologically documented infections(MDI); bloodstream infections(BSIs) (*n* = 36, 23.6%) were the most common, including central line-associated BSIs (CLABSIs) (*n* = 29, 19%). Among BSIs, 81% (29/36) were caused by Gram-negative (GN) bacteria and 19% (7/36) by Gram-positive (GP) bacteria. The most frequently isolated microorganism was coagulase-negative *Staphylococci* (*n* = 13, 8.55%), followed by *Escherichia coli* (*n* = 12, 7.89%) with a high extended-spectrum beta-lactamase (ESBL) positivity rate (69%). Fungal infections (proven+probable) were identified in 8 episodes (5.26%), and viral infections in 3 episodes (1.97%). Rabbit-derived antithymocyte globulin (RD-ATG ) was found to be significantly associated with BSIs in both univariable and logistic regression analyses (OR 8.8, 95% CI 1.692–45.761, *p* = 0.006). The attributable mortality (AM) of infection episodes was 5.2%. In multivariate analysis, bacterial BSIs, particularly GN- BSIs and those receiving RD-ATG, were significant predictors of increased mortality (*p* < 0.05). GN bacterial infections and fungal infections negatively affected survival. RD-ATG therapy was identified as a risk factor for increased infection frequency and severity, particularly in cases of BSIs and GN bacteremia. Timely and appropriate antimicrobial treatment is crucial, and the high mortality rates associated with severe infections underscore the importance of early detection and intervention.

## Introduction

 Acquired aplastic anemia (AA) is a bone marrow failure disorder characterized by a decrease in hematopoietic stem and progenitor cells, leading to pancytopenia [1]. The gold-standard treatment for children with AA is hematopoietic stem cell transplantation (HSCT) from a human leukocyte antigen (HLA)-matched sibling donor. Alternative treatment options, in the absence of a matched donor, include immunosuppressive therapy (IST) with antithymocyte globulin (ATG) and cyclosporine A (CsA) [2–4].

In febrile neutropenia (FN), infections pose a life-threatening emergency. Profound, persistent neutropenia is the primary risk factor for infections, which are the leading cause of death in AA. An infectious source is identified in about 20 to 30 per cent of FN episodes [5, 6]. In many cases, bacteremia is the sole indication of infection, occurring in 10 to 25 per cent of patients [5]. Bacteria are the most prevalent pathogens causing FN, with a shift in frequency from Gram-positive(GP) to Gram-negative(GN) bacteria over the years [1]. Children with AA and fever are at a significant risk of severe infections. Invasive bacterial or fungal infections are the leading causes of death in patients with severe aplastic anemia [7, 8]. The causative agent may be GP or GN bacteria, an opportunistic pathogen, fungus, or virus, depending on factors such as the underlying disease, duration of neutropenia, comorbid conditions, the presence of invasive procedures like central venous catheters(CVCs), as well as the choice of immunosuppressive regimens, empirical antibiotics and the use of antibacterial and antifungal agents in prophylaxis. Given the rising number of multidrug-resistant organisms, it is essential to establish local epidemiological data and the prevalence of pathogens [9].

Timely administration of appropriate antimicrobial therapy is essential in the treatment of FN. A cohort study revealed that each hour of delay in starting empiric antibacterial therapy for FN patients was associated with an 18% increase in 28-day mortality [10]. Few studies have investigated the characteristics of infections in pediatric patients with AA [7, 8, 11]. This study aimed to analyze the incidence, types and outcomes of infections in children with AA during the FN episodes.

## Methods

This single-centre retrospective cohort study was conducted at the Pediatric Hematology Department of Medical School of Ege University. Pediatric patients (< 18 years old) diagnosed with AA between January 2004 and January 2024 were included. We retrospectively evaluated the medical records of 39 pediatric patients with AA. Clinical characteristics, laboratory findings including radiological, histological, and microbiological findings, medications, immunosuppressive therapies, immunomodulators [e.g., granulocyte-colony stimulating factor (G-CSF)], comorbid conditions, history of HSCT, and outcome were obtained from medical records. The rates of infections, associated pathogens, clinical characteristics, and infection outcomes were analyzed.

Patients were followed from diagnosis until the date of HSCT, death, or last follow-up, whichever occurred first. Survival and infection analyses excluded the post-HSCT period, and patients were censored at the date of HSCT. Due to the retrospective design of the study, the analysis was conducted at the patient level rather than at the level of individual infectious episodes. Although multiple febrile neutropenia episodes occurred in some patients, detailed information regarding the exact timing, microbiological attribution, and clinical classification of each episode was not consistently available for all cases. Therefore, an episode-based analysis could not be reliably performed across the entire cohort without a high risk of misclassification, and cumulative patient-level outcomes were used for all statistical analyses.

### Definitions

- The diagnosis of severe aplastic anemia (SAA) requires the presence of at least two of the following abnormalities: granulocyte count < 0.5 × 10^9/L, platelet count < 20 × 10^9/L, and an absolute reticulocyte count ≤ 40 × 10^9/L. Additionally, bone marrow biopsy cellularity should be less than 25% of normal or less than 30% hematopoietic elements.
- Very severe aplastic anemia (VSAA) is further defined by a granulocyte count < 0.2 × 10^9/L. Non-severe aplastic anemia (NSAA) is differentiated from the severe form by the presence of less severe cytopenias and hypocellular bone marrow without meeting the criteria for SAA [12].
- An episode of FN was defined as a single oral temperature of 38.3 °C or a continuous oral temperature of 38 °C for more than 1 h, along with an absolute neutrophil count of less than 500/mm³ [13].
- Episodes of FN were categorized into three groups for analysis; Clinically documented infection (CDI), microbiologically documented infection (MDI) and unexplained group(UNX).

- CDI was defined based on the identification of the infection site through clinical and/or radiological findings (such as skin-soft tissue infections, sinusitis, pneumonia, diarrhea, rash, etc.).
- MDI was defined based on microbiological examinations of samples obtained from infection areas: direct microscopic examination, culture (bacterial, mycobacterial, mycological), polymerase chain reaction (PCR)(virus panel in respiratory samples, multiplex PCR in cerebrospinal fluid sample, mycobacterial PCR or PCR in stool).
- UNX episode was defined in patients who had no clinical or radiological findings and no microbiological documentation.
- CLABSI was defined according to the 2009 criteria established by IDSA [14].
- Invasive Fungal Infection (IFI) was defined according to the revision and update of the consensus definitions of invasive fungal disease from the European Organisation for Research and Treatment of Cancer/Mycoses Study Group criteria (EORTC/MSG) [15].

### Management of FN

In patients with fever, the source of the fever is initially investigated, and empirical treatment is determined after obtaining peripheral blood and catheter blood cultures. Other cultures are performed in patients presenting with clinical findings (urine, sputum, abscess, stool, etc.) to identify the potential source of infection. If upper respiratory symptoms or flu-like symptoms were present, a throat swab was collected for multiplex PCR testing for respiratory viruses.

FN was managed empirically with piperacillin-tazobactam or meropenem in combination with amikacin. A glycopeptide was added to the initial regimen in case of hemodynamic instability and also for Gram-positive bacterial coverage in cases where an indwelling central venous catheter was present, fever persisted beyond 48 h despite antibiotic therapy, or there was clinical evidence of skin and soft tissue infection. Abdominal ultrasound (USG) and thorax computerized tomography (CT) scans were conducted in patients with persistent fever (> 96 h) when IFIs were suspected and serial serum galactomannan(GM) level was studied. IFIs were classified into proven, probable, and possible infections according to the 2020 version of the EORTC/MSG [15]. At our centre, mould-active antifungal prophylaxis with voriconazole was started in 2013. Intravenous voriconazole was the preferred treatment in the event of fever. The treatment regimen was made based on antifungal susceptibility results.

- The attributable mortality (AM) was defined as death occurring before the clearance of cultures, with no other significant cause contributing to mortality.

### The IST protocol and ATG

- Horse ATG (ATGAM)- 20 mg/kg/day IV for 5 consecutive days or Thymoglobulin (Rabbit ATG)−2.0 mg/kg/d IV once daily,1–8 days.
- Methylprednisolone: 2 mg/kg/day IV on days 1–8.
- Prednisone taper: Following an 8-day course of IV methylprednisolone. On days 9–10, 1.5 mg/kg/d PO to be divided into two equal doses.On days 11–12, 1.0 mg/kg/d PO. On days 13–14, 0.5 mg/kg/d PO and on day 15, 0.25 mg/kg/d PO to be divided into two equal doses.
- G-CSF: 5 µg/kg/day SC from day 5, continued until ≥ 2 months of transfusion independence and ANC > 1,000/mm³, then tapered based on ANC.
- Cyclosporine A: 10 mg/kg/day PO (divided twice daily) starting day 1; trough levels maintained at 100–300 ng/mL. Continued for 1 year, followed by dose reductions of 2 mg/kg every 2 weeks [16].

Initially, horse-derived ATG (HD-ATG) was not available nationally and could not be administered; in selected cases, it was individually procured from abroad. Once access barriers were resolved, HD-ATG became routinely available and was used for all eligible patients. Accordingly, rabbit ATG (RD-ATG) was primarily used until 2014, after which HD-ATG became the standard formulation due to improved national availability.

### Antimicrobial prophylaxis

As no standardized antibacterial prophylaxis protocol existed in our institution, quinolone or other antibacterial agents were not routinely administered during the study period, with the exception of Pneumocystis jirovecii pneumonia (PCP) prophylaxis using trimethoprim–sulfamethoxazole. In contrast, mold-active antifungal prophylaxis—primarily with voriconazole—was incorporated into clinical practice beginning in 2013.

### Microbiologic methods 

Only non-duplicate isolates considered to be the causative pathogens of infection from paediatric patients were included in the study. All the strains were identified to the species level with Matrix-Assisted Laser Desorption/Ionization Time-of-Flight Mass Spectrometry [MALDI-TOF MS (BioMérieux^®^)]. The antibiotic susceptibility testing of isolates was performed with the Vitek 2 automated system (bioMérieux, Marcy l’Etoile, France) and the gradient test.

## Results

### Patient characteristics

The study included 39 pediatric patients with AA. Twenty-five patients were male (64.1%), and 14 (35.9%) were female. The median age was 12,05 years (IQR : 6 to 14,94). Acute hepatitis was identified in 6 (15.4%) patients, with causes including hepatitis A (*n* = 1), Parvovirus (*n* = 1), autoimmune hepatitis (*n* = 1), and unknown (*n* = 3). Non-A, non-B, non-C, non-D hepatitis was observed in 3 (7.6%) patients following liver transplantation, while chemical exposure was detected in 2 (5.1%) patients. The remaining 28 (71.7%) patients were considered idiopathic. Median ANC was 170/mm^3^ (range, 35–3610/mm^3^). SAA was diagnosed in 8 (20.5%) patients, VSAA in 24 (61.5%) patients, and NSAA in 7 (17.9%) patients. Thirty-two patients had received IST (ATG, CsA, pred). Among the patients receiving ATG, 16 were given RD-ATG, 15 were HD-ATG, and 2 were given both RD-ATG and HD-ATG.

The median time from diagnosis to treatment was 2 (0.75–4.75) months. At the end of the third month of treatment, 17 (43.5%) patients were non-responsive (NR), followed by 11 (28.2%) in the sixth month and 8 (20.5%) in the ninth month. Twelve (30.7%) patients underwent HSCT (Table 1 and 2)

**Table 1:** Baseline characteristics of the patients

	*n*(%)
Male	25 (64.1)
Median age, years (median, IQR)	12,05 (6–14,94)
ANC, mm^3^ (median, range)	170 (35–3610)
Severity	
SAA	8(20.5)
VSAA	24 (61.5)
NSAA	7 (17.9)
IST (ATG, CsA, pred)	32 (82)
RD-ATG	16 (41)
HD-ATG	15 (38.4)
RD-ATG + HD-ATG	2 (5.2)
Aetiology	
Idiopathic	28 (71.7)
Hepatitis	9 (23)
Chemical exposure	2 (5.1)
HSCT	12 (30.7)
Crude mortality	11 (28.2)

*ANC* absolute neutrophil count, *ATG* antithymocyte globulin, *CsA* cyclosporine A, *HD-ATG* horse-derived antithymocyte globülin, *HSCT* hematopoietic stem cell transplantation, *IST* immunosuppressive therapy, *NSAA* non-severe aplastic anemia, *RD-ATG* rabbit-derived antithymocyte globulin, *SAA* severe aplastic anemia, *VSAA* very severe aplastic anemia

**Table 2:** Detailed Characteristics, treatment Courses, and outcomes of the 39 patients

Patient number	Age(years)/sex	Etiology	Classification of AA	IST*	Treatment response (3rd month)	Treatment response (6th month	Outcome
1.	14,75/F	Idiopathic	VSAA	Yes	CR	VGPR	HSCT, alive
2.	12,5/M	Hepatitis	VSAA	No	-	-	HSCT, exitus
3.	16,5/F	Idiopathic	VSAA	Yes	VGPR	CR	Secondary AML M4, worsening of pneumonia (IFI), exitus
4.	16/F	Idiopathic	NSAA	Yes	PR	PR	Exitus
5.	12/M	Hepatitis	VSAA	No	-	-	CR, alive
6.	16,5/F	Chemical exposure	NSAA	No	-	-	CR, alive
7.	3/M	Idiopathic	VSAA	Yes	NR	NR	HSCT, Worsening of pneumonia(IFI) exitus
8.	13,3/M	Hepatitis	VSAA	Yes	VGPR	VGPR	Worsening of pneumonia(IFI) hepatosplenic candidiasis, Exitus
9.	2,5/M	Idiopathic	VSAA	Yes	NR	NR	HSCT, alive
10.	8,2/M	Idiopathic	VSAA	Yes	NR	NR	*Candida parapsilosis* (CLABSI), septic shock, exitus
11.	6/M	Post kc tx, Hepatitis	VSAA	No	-	-	HSCT, alive
12.	6,3/M	Chemical exposure	VSAA	Yes	NR	NR	NR, worsening of pneumonia exitus
13.	5/F	Idiopathic	VSAA	Yes	NR	PR	Alive
14.	8,75/F	Post kc tx, Hepatitis	SAA	Yes	NR	PR	Alive
15.	9/F	Idiopathic	VSAA	Yes	NR	NR	Secondary MDS, HSCT
16.	15,5/M	Idiopathic	SAA	Yes	-	-	Pulmonary embolism, worsening of pneumonia (*E.coli* in BAL), early exitus
17.	1,2/M	Hepatitis	VSAA	Yes	PR	PR	Alive
18.	16,5/M	Idiopathic	VSAA	Yes	NR	NR	IFI+ *Pseudomonas aeruginosa*(CLABSI), Exitus in the pre-HSCT period
19.	6/M	Idiopathic	VSAA	No	-	-	HSCT, alive
20.	14,5/M	Idiopathic	NSAA	Yes	NR	NR	HSCT
21.	12,2/F	Hepatitis	VSAA	Yes	NR	PR	Alive
22.	8,9/M	Idiopathic	SAA	Yes	PR	PR	Alive, CR, Secondary PNH
23.	13,9/F	Idiopathic	VSAA	Yes	NR	NR	HSCT, relaps, exitus
24.	12,75/F	Idiopathic	NSAA	Yes	NR	NR	HSCT, alive
25.	16,7/M	Idiopathic	NSAA	Yes	NR	NR	HSCT, alive
26.	3,6/M	Idiopathic	VSAA	Yes	PR	PR	Alive
27.	14,5/F	Idiopathic	NSAA	No	-	-	HSCT, alive
28.	5,2/M	Post kc tx, Hepatitis	SAA	Yes	NR	PR	Alive
29.	17,9/F	Idiopathic	NSAA	Yes	NR	CR	CR, alive
30.	13,3/M	Idiopathic	VSAA	Yes	PR	PR	Alive
31.	7/F	Idiopathic	SAA	Yes	VGPR	CR	Alive
32.	15,5/M	Otoimmün Hepatitis	SAA	Yes	VGPR	VGPR	Alive
33.	1,25/M	Idiopathic	SAA	Yes	PR	PR	Alive
34.	6,7/M	Idiopathic	SAA	Yes	PR	PR	Alive
35.	12,1/F	Idiopathic	NSAA	No	-	-	Alive
36.	4,8/M	Idiopathic	VSAA	Yes	CR	CR	Alive
37.	16/M	Idiopathic	VSAA	Yes	NR	PR	Alive
38.	12/M	Idiopathic	VSAA	Yes	CR	VGPR	Carbapenem resistant *Klebsiella pneumoniae* (CLABSI), septic shock, exitus
39.	11/M	Idiopathic	SAA	Yes	NR	NR	HSCT planned

*BAL* Bronchoalveolar Lavage, *CLABSI* central line-associated bloodstream infection, *CR* complete response, *F* female, *HSCT* hematopoietic stem cell transplantation, *IFI* invaziv fungal infection, *IST* immunosuppressive therapy, *M* male, *MDS* Myelodysplastic syndrome, *NR* no response, *NSAA* non-severe aplastic anaemia, *PR* partial response, *SAA* severe aplastic anaemia, *TX* transplantation, *VGPR* very good partial response, *VSAA* very severe aplastic anaemia

* The IST regimen included ATG, CsA, and prednisone

### FN episodes

A total of 152 FN episodes from 34 (87.1%) patients were evaluated. Five patients did not experience an episode of FN. Median number of FNs was 4.15 (range:1–15).

61 (40.1%) FN episodes were UNX. CDI was diagnosed in 75(49.3%) of the remaining 91 episodes, 58 episodes were MDI (38.15%), and 17 (11.18%) classified as CDI + MDI.

- The most common infection was bloodstream infections (BSIs) (*n* = 36, 23.6%), with more than half of these cases involving CLABSIs (*n* = 29, 19%).
- Infections among CDI were as follows;

25(16.4%) skin-soft tissue infections(SSTI) (12 perianal abscesses), 18(11.8%) GI infections-diarrhea, 17(11.18%) lower respiratory tract infections(LRTI), 10(6.5%) upper respiratory tract infections(URTI) and 5(3.28%) urinary tract infections(UTI).

- FN episodes with MDI (*n* = 58, 38.15%) included bacterial (*n* = 47,30.9%), fungal (*n* = 8, 5.26%) and viral (*n* = 3,1.97%) infections(Table 3).

**Table 3:** Summary of FN episodes

	**n (%)**
Total FN Episodes	152 (from 34 patients, 87.1%)
Number of FN episodes, median, range	4 (1-15)
Classification	
CDI	75 (49.3)
UNX	61 (38.1)
MDI	58 (38.1)
CDI+MDI	17 (11.1)
Site of infection	
BSI	36 (23.6)
CLABSI	29 (19)
SSTI	25 (16.4)
GI	18 (11.8)
LRTI	17 (11.1)
URTI	10 (6.5)
UTI	5 (3.2)
Etiology of MDI	
Bacterial	47 (30.9)
Gram-negative bacteria	29 (19)
- *Escherichia coli*	12 (7.8)
- *Klebsiella spp.*	4 (2.6)
- *Pseudomonas aeruginosa*	4 (2.6)
- *Acinetobacter spp.*	3 (1.9)
Gram-positive bacteria	18 (11.8)
- Coagulase-negative *Staphylococci*	13 (8.5)
- *Staphylococcus aureus*	4 (2.6)
Fungal	
Proven,CLABIs,*Candida parapsilosis*	8 (5.2)
Probable	2 (1.3)
Invasive Aspergillozis	5 (3.2)
Mucormycosis	1 (0.6)
Viral	3(1.9)
EBV	2 (1.3)
SARS-CoV-2	1 (0.6)
Outcome	
PICU	20 (13.1)
Inotropic support	20 (13.1)
MV support	10 (6.5)
Attributable mortality	8 (5.2)

*BSI* bloodstream infection, *CDI *clinically documented infection, *CLABSI *central line-associated bloodstream infection, *EBV *Epstein-Barr virüs, *FN* febrile neutropenia, *GI* gastrointestinal infection, *LRTI* lower respiratory tract infection, *MDI* microbiologically documented infection, *MV* mechanical ventilation, *PICU* pediatric intensive care unit, *SSTI* skin-soft tissue infections, *UNX* unexplained, *URTI* upper respiratory tract infection, *UTI* urinary tract infection

#### Bacterial infections

Of the bacterial infections, 29(19%) were GN and 18(11.84%) were GP. Coagulase-negative *Staphylococci* (*n* = 13,8.55%) was the most frequently isolated microorganism, followed by *Escherichia coli* (*n* = 12, 7.89%), *Klebsiella* spp. (*n* = 4, 2.6%) *Pseudomonas aeruginosa* (*n* = 4, 2.6%), *Staphylococcus aureus* (*n* = 4, 2.6%) and *Acinetobacter* spp.(*n* = 3, 1.97%).

**Fig. 1:**
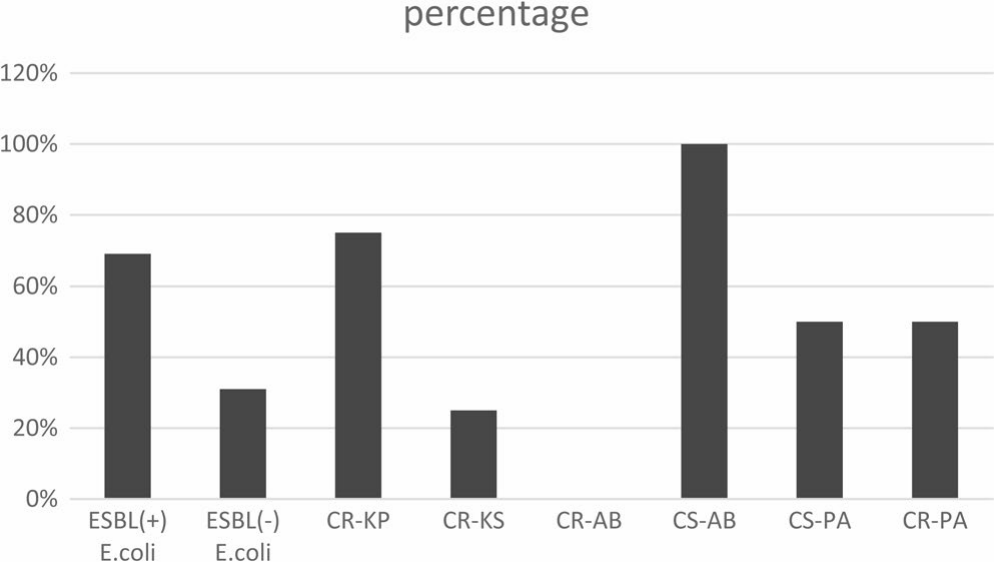
Resistance spectrum of Gram-negative microorganisms. Abbreviations:CS-AB, Carbapenem-susceptible *Acinetobacter baumannii; *CR-ABCarbapenem-resistant* Acinetobacter baumannii ; *CS-KP*,* Carbapenem-susceptible *Klebsiella pneumoniae*; CR-KP, Carbapenem-resistant* Klebsiella pneumoniae* ; CS-PA, Carbapenem-susceptible *Pseudomonas aeruginosa*; CR-PA, Carbapenem-resistant* Pseudomonas aeruginosa; *ESBL*,*Extended-spectrum beta-lactamase

*Escherichia coli* was the most commonly isolated GN organism, with a high extended-spectrum beta-lactamases (ESBL) positivity rate (9/12, 69%). Carbapenem resistance (CR) was observed in 3 (1.97%) episodes with *Klebsiella* spp.(3/4, 75%). No CR was detected among *the Acinetobacter* species (Fig. 1).

**Fig. 2:**
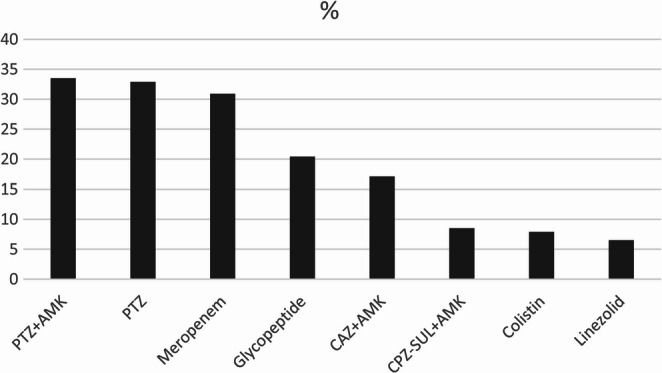
Distribution of antimicrobials used during febrile neutropenia attacks. Abbreviations:AMK,amikacin; CAZ, ceftazidime; CPZ-SUL, cefoperazone–sulbactam (sulperazone); PTZ, piperacillin–tazobactam

The distribution of antimicrobials used during febrile neutropenia attacks is shown in Fig. 2.

#### Fungal infections

A total of 2 proven and 6 probable invasive fungal infections (IFIs) (5.26%) were diagnosed. The proven IFIs were CLABIs caused by *Candida parapsilosis*, which led to the removal of the catheter.

Five episodes of probable invasive Aspergillosis (IA) were diagnosed. In four of these, consecutive blood GM antigen positivity was noted, along with clinical and radiological findings. In another episode, *Aspergillus fumigatus* was isolated from sputum, supported by clinical and radiological findings.

On radiology, a patient with periorbital cellulitis and fungal sinusitis was diagnosed with probable mucormycosis based on the observation of hyphal structures in both the sinus aspirate and tissue sample, which provided microbiological evidence.

Our centre started routinely using voriconazole for antifungal prophylaxis in 2013. Since then, voriconazole prophylaxis has been administered to 19 (48.7%) of the patients. When comparing both periods in terms of IFI incidence, although the number of cases decreased after 2013 (*n* = 4/20 vs. *n* = 1/19), the difference was not statistically significant (*P* = 0.57, OR: 0.188, 95% CI: 0.024–2.336).

#### Viral Infections

Viral infections were identified in 3 (1.97%) episodes: one was diagnosed with SARS-CoV-2 via PCR on a nasopharyngeal swab, despite the absence of signs of LRTI. At the same time, the other two had self-limiting EBV viremia that did not require treatment.

### Risk factors

In the univariate analysis, variables significantly associated with BSIs were evaluated. No statistically significant differences were observed concerning age, gender, ANC, or whether ATG was administered. However, the type of ATG received—specifically RD-ATG was found to be significantly associated with BSIs in both univariable and logistic regression analyses. RD-ATG was identified as an independent risk factor for BSIs (OR 8.8, 95% CI 1.692–45.761, p = 0.006) (Table 4).

**Table 4:** Risk factors for BSIs

	Patients with BSI *N* = 20	Non-BSI *N* = 19	*p*-value(95% CI)
Age, months, median, range	106 (14–200)	151 (29–214)	0.57
Sex, female	8 (40%)	6 (31.5%)	0.46 (0.354–5.026)
ANC, mm3,median, range	163 (0–2360)	193 (20–3610)	0.50
Receiving ATG	13 (68.4%)	14 (77.8%)	0.52 (0.142–2.701)
RD-ATG	12 (70.6%)	3 (21.4%)	**0.006** **(1.692–45.761)**

*ANC* absolute neutrophil count, *ATG* anti-thymocyte globulin, *BSI* bloodstream infection, *RD-ATG* rabbit-derived anti-thymocyte globulin, *SAA* severe aplastic anemia

#### Role of ATG

FN occurred in 30 out of 33 patients who received ATG. There was no statistically significant difference in the incidence of FN episodes between patients receiving HD or RD- ATG (p=0.44, 95% CI: 0.032–4.960).

In the univariate analysis, a comparison of ATG recipients revealed that those receiving RD-ATG experienced a higher frequency of FN episodes, MDI, BSI, GN bacteremia, IFI, and septic shock. Statistically significant differences were identified, with BSI (p=0.006, 95%CI 1.692-45.761), GN BSI (p=0.048, 95% CI 0.972-20.827) and mortality rate (p=0.024, 95%CI 0.010-0.949) being notably more prevalent among RD-ATG recipients (Table 5).

**Table 5:** Evaluation of FN episodes among ATG recipients

	HD-ATG (*n* = 16)(%)	RD-ATG (*n* = 15) (%)	*p*-value (95% CI)
Number of FN, median, range	2 (0–15)	4(1–18)	0.49
FN episodes > 5	5 (31.3)	9 (60)	0.10 (0.069–1.329)
MDI	8 (50)	12 (80)	0.08 (0.807–19.810)
BSI	5 (31.3)	12 (80)	**0.006 (1.692–45.761)**
GN BSI	4 (25)	9 (60)	**0.048 (0.972–20.827)**
Fungal infection	1 (6.3)	4 (26.7)	0.122 (0.533–55.800)
Septic shock	3(18.8)	5 (33.3)	0.35 (0.415–11.302)
Mortality	1(7.1)	7(43.8)	**0.024 (0.010–0.949)**

*BSI* bloodstream infection, *FN* febrile neutropenia, *GN-BSI* Gram-negative bloodstream infection, *HD-ATG* horse-derived anti thymocyte globulin, *MDI* microbiologically documented infection, *RD-ATG* rabbit-derived anti thymocyte globulin

### Outcome and attributed mortality

Among all episodes, 20 patients (13.1%) required admission to the intensive care unit (ICU) and inotropic support due to septic shock, with half of these patients (n=10, 6.5%) also necessitating mechanical ventilation(MV). Eight patients died during the episode, resulting in an AM rate of 5.26%.

Five patients experienced a worsening of pneumonia symptoms. In two of these cases, the causative agent remained unidentified. In one patient, *Escherichia coli* was detected in bronchoalveolar lavage. The remaining three patients were diagnosed with fungal pneumonia, one of whom also had hepatosplenic candidiasis. However, the causative agent could not be identified in any of these fungal pneumonia cases.

Additionally, three patients died due to septic shock caused by GN bacteria associated with CLABSIs. The identified pathogens were *Candida parapsilosis* in one patient, *Pseudomonas aeruginosa* in another, and carbapenem-resistant *Klebsiella pneumoniae* in the third. Despite prompt catheter removal and intensive care interventions, all three patients succumbed to their infections. Notably, the patient who died from *Pseudomonas* sepsis also had concurrent fungal pneumonia, confirmed by a positive GM test in bronchoalveolar lavage and consistent radiological findings.

Considering the introduction of horse ATG and the routine implementation of mold-active antifungal prophylaxis after 2014, as well as the long 20-year duration of the study period, the cohort was stratified into two intervals: before and after 2014. When these periods were compared, univariate analysis showed that there were no statistically significant differences in the rates of MDI, BSI, UNX, IFIs, GN infections, or mortality (p > 0.05). In contrast, the rate of CLABSI was lower in the post-2014 period, and a statistically significant difference was identified (p = 0.026; 95% CI, 1.021–12.600).

Comparison of outcomes between SAA/VSAA and NSAA patients revealed no significant differences in MDI ( p=0,412; 95% CI, 0.126–2.955), BSI ( p=0,654; 95% CI, 0.162-7.552) , CLABSI ( p=0,567; 95% CI, 0.088-5.578), IFI( p=0,452; 95% CI, 0.212–23.832) , PICU support ( p=0,671; 95% CI, 0.041–12.347) , and mortality ( p=0,245; 95% CI, 0.454–15.162) rates.

In univariate analysis, mortality was significantly higher in patients who did not receive mold-active antifungal prophylaxis (p=0.022, 95%CI 0.110-0.944), those treated with RD-ATG (p=0.024, 95%CI 0.010-0.949), and those who were non-responsive (NR) to IST treatment in the third month (p=0.021, 95%CI 0Ç435-0.950). Sex and age did not have a significant effect on the mortality rate (p> 0.05).

In multivariate analysis, BSIs were associated with increased mortality (p=0.05, OR: 9.154, 95% CI: 0.998–83.996). Specifically, GN-BSIs showed a significant association with higher mortality (p=0.04, OR: 6.000, 95% CI: 1.021–35.268). Additionally, receiving RD-ATG was linked to increased mortality (p=0.04, OR: 10.111, 95% CI: 1.054–97.002) (Table 6).

**Table 6:** Multivariate analysis of risk factors for mortality

Age, months	*P* value	OR (95%CI)
	0.49	1.00 (0.991–1.019)
Sex	0.15	0.198 (0.022–1.809)
ANC	0.21	0.999 (0.997–1001)
Receiving ATG	0.27	3.500 (0.377–32.503)
Receiving RD-ATG	**0.04**	**10.111 (1.054–97.002)**
Lack of antifungal prophylaxis	**0.04**	**9.692 (1.060–88.653.060.653)**
BSI	**0.05**	**9.154 (0.998–83.996)**
GN BSI	**0.04**	**6.000 (1.021–35.268)**
Septic shock	0.06	5.00 (0.926–26.990)

*ANC* absolute neutrophil count, *ATG* anti-thymocyte globulin, *BSI* bloodstream infection, *GN-BSI* Gram-negative bloodstream infection, *RD-ATG* rabbit-derived anti-thymocyte globulin, *SAA* severe aplastic anemia

**Fig. 3:**
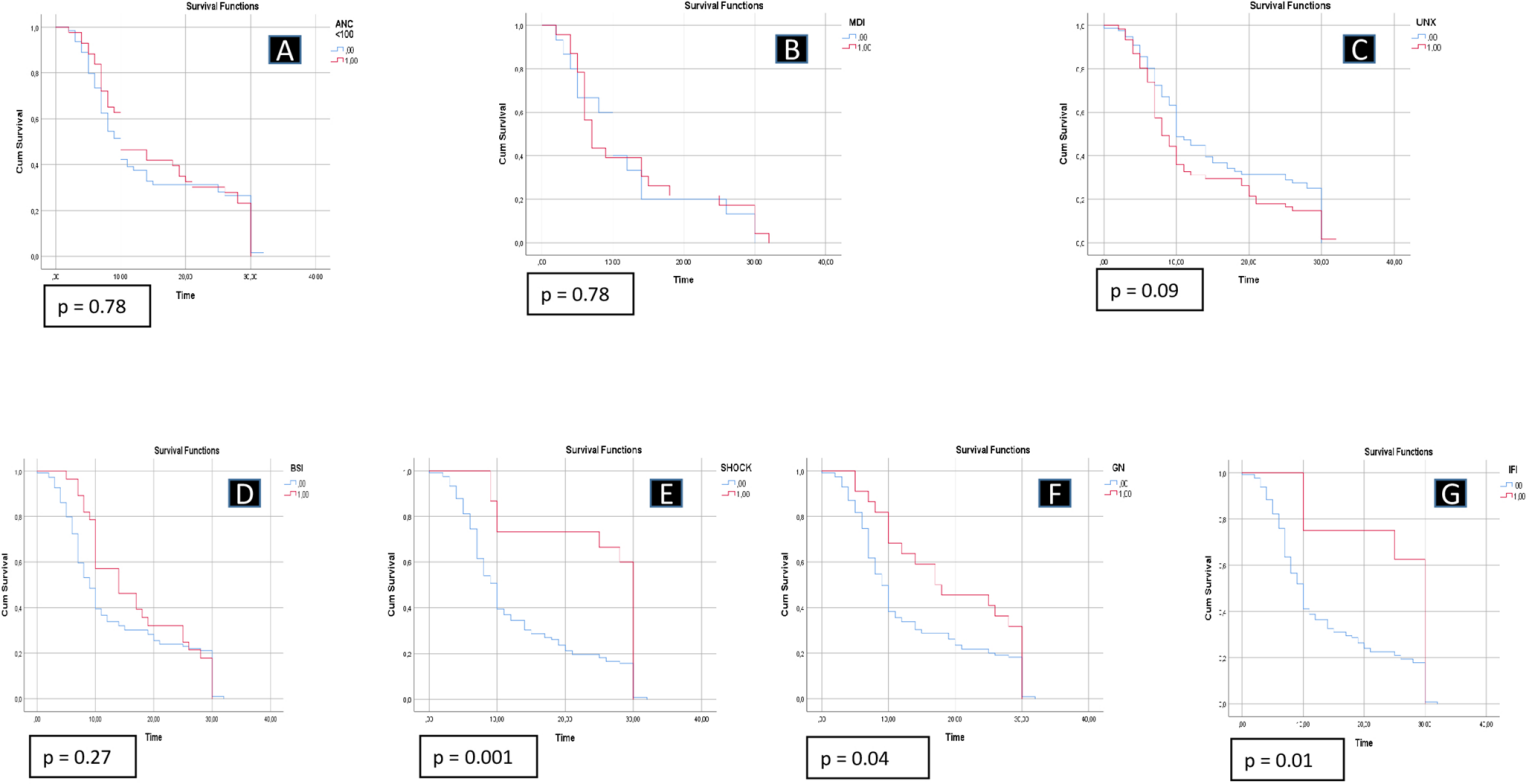
Survival in pediatric aplastic anemia patients with febril neutropenia episodes. (**a**) ANC, Absolute neutrophil count; (**b**) MDI, microbiologically documented infection; **c**) UNX, unexplained episode; d) BSI, bloodstream infection; **e**) CLABSI, Central Line–Associated Blood Stream Infections; **f**) septic shock; **g**) GN, Gram-negative infection; **h**) IFI, invasive fungal infection

In our study, survival was not associated with the ANC during infection episodes. Our analysis revealed no significant differences in survival outcomes among various infection types (MDI, BSI, CLABSI, UNX). However, experiencing GN bacterial infection and fungal infections had a negative impact on survival (Fig. 3).

## Discussion

This study evaluated the incidence, types, risk factors and outcomes of infections during FN episodes in pediatric patients with AA. Our findings indicate that infections are a significant cause of morbidity and mortality in this vulnerable patient population. The overall incidence of FN episodes was high, with 87.1% of patients experiencing at least one episode. CDIs were present in 49.3% of cases, while MDIs were identified in 38.1%. Additionally, UNX episodes were observed in 40.1% of patients.

Studies on the epidemiology of infections during FN episodes in individuals with AA are limited, with particularly scarce data available on FN in pediatric AA patients. Similarly to our study, Samanta et al. [9] reported that infectious foci were identified in 68.4% of the episodes, while no focus could be identified in the remaining 31.6% of episodes. Of those, CDIs were identified in 55.2%, MDI in 39.47% episodes, whereas bacteremia was seen in 34.2% of the episodes. Comparable rates have been documented in previous studies, including those involving adult patient groups [17, 18]. Although previous studies identified LRTIs as the most common site among CDIs, SSTIs were the most prevalent, with a notable frequency of perianal abscesses in our series [1, 7, 9].

The most common infection was BSIs (23.6%) in MDI group, with a high rate of CLABSIs (19%), predominantly caused by GN bacteria in our study. Notably, *Escherichia coli* was the most commonly isolated GN pathogen, exhibiting a high rate of ESBL positivity (69%). Several studies have reported bacteremia in 20–38% of FN episodes in children with SAA [1, 5, 7]. Quarello et al. [1] reported that bacteremia was the most common type of documented infection, accounting for 38% of episodes, similar to our study. However, in contrast to our findings, GP microorganisms were more frequently identified. The ratio of GP to GN bacteria may fluctuate over time and vary across centres due to multiple factors, including the use of prophylactic antibiotics, institutional antibiotic preferences and prescribing habits, catheter utilization practices, and surveillance protocols. The literature also provides data supporting this. Torres et al. [7] reported that GP bacteria accounted for 76% of all bacteria, while Lertpongpiroon et al. [17] reported that common pathogens identified were GN bacteria (52.9%), including *Acinetobacter baumannii* and *Pseudomonas aeruginosa*, in a cohort that also included adults with AA. The high rate of multidrug-resistant (MDR) GN infections (particularly ESBL-producing *Escherichia coli* and carbapenem-resistant *Klebsiella pneumoniae*) is concerning and aligns with global trends observed in pediatric patients with hematologic malignancies and bone marrow failure syndromes [19, 20]. This shift towards MDR organisms underscores the urgent need for tailored antimicrobial stewardship programs and optimized empirical therapy strategies in high-risk pediatric hematology units.

Fungal infections were detected in 5.26% of FN episodes, with *Candida parapsilosis* and *Aspergillus* species being the primary isolates. Higher rates of IFIs were reported in previous studies; the IFI rate was 21.7% and all were *Aspergillus* spp in a study [16]. The incidence of IFIs was 11%, and invasive fungal pneumonia was the most common site of infection (69.2%) in another study [18]. Probable invasive Aspergillosis (IA) constituted the majority of IFIs in our series. The introduction of mould-active antifungal prophylaxis (voriconazole) in 2013 in our centre was associated with a trend towards a decrease in IFIs, although this reduction did not reach statistical significance.

FN was identified as the most common adverse event, affecting 57.1% of patients, with infection, pneumonia, and sepsis being the primary causes of mortality following IST and ATG treatment [21].

A statistically significant association between the grade of aplasia at diagnosis and the incidence of infection episodes was determined in a recent study [1]. HD-ATG is recommended as first-line immunosuppressive therapy in AA as RD-ATG has been shown to be inferior as initial treatment in terms of hematologic response and survival, and is therefore generally reserved for selected clinical settings or refractory cases [22, 23]. Because of limited national availability in the early study period, HD-ATG could not be routinely used and RD-ATG was predominantly administered until 2014; after access barriers were resolved, HD-ATG became routinely available and replaced RD-ATG as the standard therapy. Our study identified an association between the use of RD-ATG and an increased risk of infections. Patients treated with RD-ATG exhibited a greater incidence of FN episodes, MDI, BSI, GN bacteremia, IFI, and septic shock. Notably, the rates of BSIs, GN bacteremia, and IFIs were significantly higher in patients receiving RD-ATG compared to those treated with HD-ATG. This finding is consistent with previous research indicating that RD-ATG is associated with prolonged and profound immunosuppression. These results highlight the importance of close monitoring and early intervention for patients undergoing RD-ATG-based immunosuppressive therapy. GN sepsis and fungal infections were common and were identified as leading causes of mortality during the post-ATG period in previous studies [18, 24].

The literature has highlighted that infections are the leading cause of death in patients with AA [7, 8, 17, 25]. An unfavourable outcome was reported in 42% of patients with FN, with 54% developing bacteremia and 30.7% being diagnosed with IFIs [9]. In another series, the mortality rate attributed to an infection episode in children was reported as 9% [1]. In this cohort, the AM rate of infections during FN episodes was 5.26% and was primarily attributed to severe bacterial infections, such as GN BSIs and septic shock. Multivariate analyses revealed that BSIs (OR: 9.154, 95% CI: 0.998–83.996, *p* = 0.05) and GN BSIs (OR: 6.000, 95% CI: 1.021–35.268, *p* = 0.04) were significantly associated with mortality. Survival was not associated with the ANC during infection episodes and infection types. However, the presence of GN bacterial infections and fungal infections negatively impacted survival. Lionel et al. [18] reported that pediatric AA, very severe aplastic anaemia, pre or post-ATG infections, and lack of response to ATG were the significant predictors of mortality, and mortality was highest in patients with combined bacterial and fungal infections post-IST.

These findings reinforce the critical importance of early diagnosis and prompt initiation of effective antimicrobial therapy. Previous studies have demonstrated that delays in administering appropriate antibiotics are associated with increased mortality in febrile neutropenic patients [10, 26, 27]. Therefore, implementing rapid diagnostic techniques and optimizing empiric treatment protocols are crucial in managing pediatric AA patients.

### Limitations of the study

This study has several limitations. First, its retrospective single-center design may limit the generalizability of the findings and introduces the possibility of information and selection bias. An important limitation of the present study is the inability to perform an episode-based analysis of febrile neutropenia. Although several patients experienced multiple infectious episodes, the retrospective nature of the dataset and incomplete documentation of episode-specific clinical and microbiological details prevented a robust and standardized episode-level evaluation. As a result, our analyses reflect cumulative patient-level outcomes rather than the full temporal heterogeneity of individual infectious events. This approach may have masked within-patient variability and limited the assessment of dynamic changes in infection patterns over time. Future prospective studies with standardized episode-level data collection are needed to better characterize the clinical course and outcomes of recurrent febrile neutropenia in this population.The relatively small sample size, inherent to the rarity of pediatric acquired aplastic anemia, may have reduced the statistical power of subgroup analyses, particularly in evaluating predictors of invasive fungal infections and mortality. Due to the long 20-year study period, changes in diagnostic techniques, antimicrobial stewardship practices, supportive care strategies, and prophylaxis protocols—including the introduction of mold-active antifungal prophylaxis after 2013—may have introduced heterogeneity in clinical management. Additionally, detailed data on the duration and depth of neutropenia, which represent key determinants of infection risk, were not uniformly available for all FN episodes and therefore could not be fully incorporated into the multivariable models. The choice of RD-ATG versus HD-ATG was influenced by drug availability rather than standardized indications, which may have confounded the observed associations. Finally, microbiological documentation was limited in part by culture-negative episodes and evolving diagnostic modalities, potentially leading to underestimation of viral or fungal infections. Despite these limitations, the study provides valuable longitudinal data on infection epidemiology and clinical outcomes in pediatric AA patients. Our findings emphasize the importance of proactive infection control measures, timely antimicrobial therapy, and individualized prophylactic strategies. Further research is needed to optimize infection prevention and treatment protocols to improve clinical outcomes in this high-risk group.

In conclusion, infections remain a major cause of morbidity and mortality in pediatric AA patients, with BSIs, GN bacteremia, and fungal infections posing the greatest risks. RD-ATG therapy is associated with significantly higher infection-related mortality, necessitating closer monitoring and tailored prophylactic strategies. Survival was not significantly correlated with the severity of neutropenia; however, the presence of GN bacterial infections and IFIs was associated with poorer survival outcomes. Strengthening infection prevention measures, optimizing empirical antimicrobial regimens, and integrating multidisciplinary infection management protocols are essential to improving clinical outcomes in this high-risk population.

## Data Availability

The datasets generated and/or analyzed during the current study are available from the corresponding author on reasonable request.
